# Gas channels and CO_2_ transport across cell membranes: Mechanisms and synergy

**DOI:** 10.1113/JP290205

**Published:** 2025-12-20

**Authors:** Nazih L. Nakhoul

**Affiliations:** ^1^ Department of Medicine Section of Nephrology Tulane Medical School New Orleans Louisiana USA

**Keywords:** AQP5, carbonic anhydrase II, carbonic anhydrase IV

## CO_2_ transport across cell membranes

The transport of gases, such as CO_2_, NH_3_ and O_2_, across cell membranes is critical for all forms of life. It was generally accepted that gases freely and passively cross the cell membrane barrier governed by the well‐established solubility‐diffusion model. As early as 1830 it was established that *solubility* across membranes increases with the lipophilic nature of the molecule. Shortly thereafter the principle of *‘diffusion’* was introduced to the solubility concept. In 1855 Fick's theory of diffusion established the parameters that govern the flow of solutes. Extending the solubility‐diffusion model to include membrane barriers, the rate of transport of CO_2_ across a cell membrane can be expressed as *J*_CO2_ = *P*_CO2_. ([CO_2_]o – [CO_2_]i), where *J*_CO2_ is the flux of CO_2_ and *P*_CO2_ is the permeability of CO_2_ driven by the concentration gradient of CO_2_ across the membrane. *P*_CO2_ includes the diffusion coefficient of CO_2_ (*D*_CO2_) and the area of CO_2_ exposure and is inversely related to the barrier thickness (bulk solution and cell membrane).

Around 1900 Meyer and Overton, working independently on anaesthetics, recognized an association between the lipid solubility of substances and their increased ability to cross the membrane. This association, known as the Meyer–Overton rule, is inaccurately often used to mean the solubility‐diffusion model.

## Control of CO_2_ diffusion across cellular membranes

Building on the principles of diffusion, a cell can influence membrane transport of CO_2_ by controlling *membrane permeability* or the *CO_2_ concentration gradient* across the cell membrane. Membrane permeability can be affected by altering the composition of the lipid bilayer or by the presence of membrane proteins. Abundance of the hydrophilic membrane proteins, which do not conduct CO_2_, and their arrangement can usually hinder or obstruct CO_2_ access to the membrane.

On the other hand, maintaining the CO_2_ concentration gradient is dependent on CO_2_ build‐up in unstirred layers adjacent to the membrane. When a cell is exposed to CO_2_‐containing solution, it diffuses through the extracellular unstirred layer, through the membrane and then through an intracellular layer before moving to the intracellular compartment. Accumulation of CO_2_ in the membrane intracellular unstirred layer will reduce the concentration gradient and slow diffusion of CO_2_. Converting intracellular CO_2_ to HCO_3_^−^ will keep low intracellular CO_2_ concentration adjacent to the membrane. Similarly CO_2_ in the extracellular unstirred layer needs to be replenished from HCO_3_^−^ to maintain the gradient. Interconversion of CO_2_ and HCO_3_^−^ is a slow process that is highly accelerated by carbonic anhydrases (CAs).

## Gas channels and CO_2_ transport

The widespread acceptance of Overton's rule that all gases cross all cell membranes by dissolving in the lipid layer of the cell membrane was challenged by two important discoveries. Boron's group in 1994 (Waisbren et al., 1994) reported that specific cell membranes were relatively impermeable to CO_2_. This was followed by another seminal discovery that the water channel AQP1, expressed in oocytes, increased the permeability of the membrane to CO_2_, thus acting as a CO_2_ channel (Nakhoul et al., 1998). Using pH microelectrodes to measure intracellular pH, exposure of the oocyte to a solution containing CO_2_/HCO_3_^−^ caused intracellular acidification due to the intracellular reaction: CO_2_ + H_2_O↔HCO_3_^−^ + H^+^. As such CO_2_ transport was estimated from the pH_i_ decrease and the rate of intracellular acidification. Because hydration of CO_2_ is slow, build‐up of CO_2_ in the intracellular unstirred layer could decrease the concentration gradient of CO_2_. Indeed injecting the oocyte with CA increased CO_2_ influx significantly in oocytes expressing AQP1 compared to H_2_O‐injected oocytes.

## Synergistic mechanisms

This narrative briefly describes the important hallmarks of studying CO_2_ transport across cell membranes. The important points include (i) the permeability and diffusion of CO_2_, (ii) the role of CAs and (iii) the AQPs as CO_2_ channels. Boron's group has contributed significantly to elucidating the mechanisms of all these points. In those studies identifying cell membranes that are impermeable to CO_2_ led to the discovery of AQP1 as a CO_2_ channel. In a series of several elegant papers, they examined the role of CA in affecting CO_2_ transport. In the first paper (Musa‐Aziz et al., 2014a) they addressed the role of intracellular CA. They showed that injecting oocytes with soluble CA‐II accelerated CO_2_ influx and this influx was blocked by ethoxzolamide. In the second paper (Musa‐Aziz et al., 2014b) they examined the effect of extracellular CA by expressing CA‐IV at the cell membrane. The same group then used mathematical modelling to interpret the CO_2_ influx in the oocyte.

The paper in this issue by Wang et al. (2025) examines all three factors that were previously studied independently to address CO_2_ membrane transport. They systematically assessed functional interactions among AQP5 (a water channel expressed in the lung), intracellular CA and extracellular CA. They used demanding simultaneous measurements of intracellular and membrane‐surface pH to measure CO_2_ transport in presence and absence of AQP5. This approach made it possible to determine the response in CO_2_ transport when changes and measurements were made on the same side of the membrane (e.g. intracellular CA‐II and intracellular pH) named ‘cis measurements’ or on opposite sides of the membrane (e.g. CA‐II and surface pH) named ‘trans measurements’. The results of the study demonstrated independent cooperation among intracellular and extracellular CA and AQP5, which does not involve direct physical interaction but augmented CO_2_ transport to more than an additive effect in what is termed ‘synergistic’ effect. These findings are novel and provide important insights into the factors that govern CO_2_ transport across cell membranes.

## Additional information

### Competing interests

No competing interests declared.

### Author contributions

N.L.N.: conception or design of the work; drafting the work or revising it critically for important intellectual content; final approval of the version to be published; agreement to be accountable for all aspects of the work.

### Funding

No funding.

## Supporting information


Peer Review History
